# Multi-omics and experimental analysis unveil theragnostic value and immunological roles of inner membrane mitochondrial protein (IMMT) in breast cancer

**DOI:** 10.1186/s12967-023-04035-4

**Published:** 2023-03-10

**Authors:** Hung-Yu Lin, Hsing-Ju Wu, Pei-Yi Chu

**Affiliations:** 1 Department of Post-Baccalaureate Medicine, College of Medicine, National Chung Hsing University, Taichung, 402 Taiwan; 2 Research Assistant Center, Show Chwan Memorial Hospital, Changhua, 500 Taiwan; 3 Department of Biology, National Changhua University of Education, Changhua, 500 Taiwan; 4 School of Medicine, College of Medicine, Fu Jen Catholic University, New Taipei City, 242 Taiwan; 5 Department of Pathology, Show Chwan Memorial Hospital, Changhua, 500 Taiwan; 6 Department of Health Food, Chung Chou University of Science and Technology, Changhua, 510 Taiwan; 7 National Institute of Cancer Research, National Health Research Institutes, Tainan, 704 Taiwan

**Keywords:** Breast cancer, Inner membrane mitochondrial protein, Diagnostic biomarker, Prognosis, Precision medicine, Tumor immune microenvironment

## Abstract

**Background:**

The inner membrane mitochondrial protein (IMMT) is a central unit of the mitochondrial contact site and cristae organizing system (MICOS). While researchers continue to demonstrate the physiological function of IMMT in regulating mitochondrial dynamics and preserving mitochondrial structural integrity, the roles of IMMT in clinicopathology, the tumor immune microenvironment (TIME), and precision oncology in breast cancer (BC) remain unclear.

**Methods:**

Multi-omics analysis was used here to evaluate the diagnostic and prognostic value of IMMT. Web applications aimed at analyzing the whole tumor tissue, single cells, and spatial transcriptomics were used to examine the relationship of IMMT with TIME. Gene set enrichment analysis (GSEA) was employed to determine the primary biological impact of IMMT. Experimental verification using siRNA knockdown and clinical specimens of BC patients confirmed the mechanisms behind IMMT on BC cells and the clinical significance, respectively. Potent drugs were identified by accessing the data repositories of CRISPR-based drug screenings.

**Results:**

High IMMT expression served as an independent diagnostic biomarker, correlated with advanced clinical status, and indicated a poor relapse-free survival (RFS) rate for patients with BC. Although, the contents of Th1, Th2, MSC, macrophages, basophil, CD4 + T cell and B cell, and TMB levels counteracted the prognostic significance. Single-cell level and whole-tissue level analyses revealed that high IMMT was associated with an immunosuppressive TIME. GSEA identified IMMT perturbation as involved in cell cycle progression and mitochondrial antioxidant defenses. Experimental knockdown of IMMT impeded the migration and viability of BC cells, arrested the cell cycle, disturbed mitochondrial function, and increased the ROS level and lipid peroxidation. The clinical values of IMMT were amenable to ethnic Chinese BC patients, and can be extrapolated to some other cancer types. Furthermore, we discovered that pyridostatin acted as a potent drug candidate in BC cells harboring an elevated IMMT expression.

**Conclusion:**

This study combined a multi-omics survey with experimental verification to reveal the novel clinical significance of IMMT in BC, demonstrating its role in TIME, cancer cell growth and mitochondrial fitness, and identified pyridostatin as a promising drug candidate for the development of precision medicine.

**Supplementary Information:**

The online version contains supplementary material available at 10.1186/s12967-023-04035-4.

## Background

According to a status report on the global cancer burden provided by GLOBOCAN 2020, breast cancer (BC) is the most commonly diagnosed cancer among women, while it is the fourth leading cause of cancer deaths globally [1]. Based on the presence or absence of biomarkers for estrogen receptors, progesterone receptors and human epidermal growth factor 2 (HER2), BC can be categorized as luminal A/B, HER2-positive, or triple-negative breast cancer (TNBC) [2]. To date, surgery, radiation, and endocrine therapy remain the primary therapeutic strategies to manage BC [3]. Neoadjuvant therapies, including chemotherapy combined with targeted agents have been widely applied in BC patients with high recurrence and metastasis [4]. Unfortunately, a majority of patients develop resistance to these treatments and inevitably relapse [5]. Thus, identifying reliable and accurate biomarkers for the diagnosis, prognosis, and therapeutic targeting of BC is imperative.

The primary cause of death in BC patients is cancer metastasis, associated with metabolic reprogramming which cultivates a corrupted tumor microenvironment, thereby counteracting therapy-induced cell death [6]. Mitochondria are dynamic organelles which provide metabolic support and regulate cellular functions, including calcium homeostasis, redox status, and programmed cell death. Mounting evidence indicates that tumor cells modify mitochondrial dynamics to enhance proliferation and survival, thus the targeting of mitochondrial dynamics may be an effective strategy to suppress the metastatic ability of BC cells [7].

The predominant role of the mitochondrial contact site and cristae organizing system (MICOS) complex in structuring the inner mitochondrial membrane (IMM) and cristae junctions formation and in affecting mitochondrial dynamics and metabolism has recently become the focus of a growing number of investigations [8]. The MICOS complex is comprised of MIC10/MICOS10 (mitochondrial contact site and cristae organizing system subunit 10), MIC13/QIL1 (mitochondrial contact site and cristae organizing system subunit 13), MIC19/CHCHD3 (coiled-coil-helix-coiled-coil-helix domain containing 3), MIC25/CHCHD6 (coiled-coil-helix-coiled-coil-helix domain containing 6), MIC26/APOO (apolipoprotein O), MIC27/APOOL (apolipoprotein O like), and MIC60/IMMT (inner membrane mitochondrial protein, also known as mitofilin) [8]. IMMT is generally recognized as the core of MICOS [9, 10], while the downregulation, modification or destruction of IMMT may lead to mitochondrial dysfunction, and ultimately cell death. In addition, IMMT is a hallmark of various diseases, including cancer [10]. However, the role of *IMMT* in clinicopathology, the tumor immune microenvironment (TIME), and precision oncology remain unclear.

The integrated analysis we applied in this study aimed to investigate the theragnostic value of IMMT in BC. We first examined the differential expression, diagnostic efficacy, and prognostic value of IMMT. Second, the association between IMMT expression and TIME at the single-cell and whole-tissue levels were determined. Third, the possible mechanism underlying the tumor-promoting role of IMMT was identified by functional enrichment modules and confirmed by experimental verification. Fourth, we confirmed the clinicopathological significance of IMMT using real-world ethnic Chinese BC patients’ tissues and by pan-cancer bioinformatics. Finally, by accessing the Genomics of Drug Sensitivity in Cancer (GDSC) and Cancer Cell Line Encyclopedia (CCLE) cell data repositories, we identified pyridostatin as an effective drug candidate for cancer cells with high IMMT expression.

## Materials and methods

### Gene differential expression and prognostic significance

Data from TNMplot [11], UALCAN (The University of ALabama at Birmingham CANcer data analysis Portal) [12], Breast Cancer Gene-Expression Miner (bc-GenExMiner) v4.8 [13], and Gene Expression Omnibus (GEO) were used to analyze the gene expression levels in BC tumors and adjacent normal tissues. The Human Protein Atlas [14–16] was accessed to determine immunohistochemical (IHC) staining images. The Kaplan–Meier plotter [17] was applied to analyze the survival rates associated with the various clinical stages of BC, wherein the patient groups were divided by “Auto select best cut off”, thereby selecting the best-performing cutoff value.

### Single-cell and immune analyses

The Tumor Immune Single Cell Hub (TISCH) was utilized to conduct single-cell analyses [18] to determine which BC cell types may express IMMT. Next, independent datasets from the scTIME Portal [19], consisting of BC patients’ cells, were analyzed on GSE75688 and visualized in UMAP. A heatmap was used to illustrate the signature expression of Mitophagy in GSE75688. The cellular communication among various T cell subsets was analyzed by the LR network of the scTIME Portal. SpatialDB was used to analyze the spatial transcriptomics [20], whereby the gene expression in tissue sections can be visualized and quantified. The IMMT expression in the immune cells of BC tissue was determined based on the GSE114724 dataset. TISIDB was used to conduct the Spearman correlation test for IMMT expression with immune infiltration levels [21]. GEPIA2 was used to determine the correlation of IMMT with the immune cell signature [22]. Estimation of Stromal and Immune cells in Malignant Tumor tissues using Expression data (ESTIMATE) was used to evaluate the matrix content, immune cell infiltration levels, comprehensive score, and tumor purity [23].

### Genetic and enrichment analyses

The GSCALite platform was employed to analyze the single nucleotide variants (SNVs) [24]. The TIMER platform was employed to analyze the Pearson correlation coefficient of mutation with gene expression in TCGA samples [25–27]. The UCSC Xena portal and DriverDBv3 were used to evaluate the correlation of copy number with gene expression in TCGA samples [28, 29]. The LinkedOmics platform was used to identify IMMT co-expressed genes and conduct a gene set enrichment analysis (GSEA) to illustrate the Reactome pathway [30].

### Cell culture and transfection

The cell culture and transfection were conducted as previously described [31]. Briefly, MDA-MB-231 cells were grown in DMEM (HyClone) supplemented with 10% fetal bovine serum (Thermo Fisher Scientific). The existence of mycoplasma was determined using the e-Myco™ plus Mycoplasma PCR Detection Kit (iNtODEWORLD, MA, USA). For the knockdown of IMMT, 20 nM of IMMT-targeting siRNA (4392420, Thermo Fisher Scientific) and its corresponding negative control RNA (4390843, Thermo Fisher Scientific) were introduced into cells using the Lipofectamine™ RNAiMAX Transfection Reagent (LMRNA015, Invitrogen, Carlsbad, CA, USA). The siRNA transfection efficiency was determined by western blot 72 h after transfection.

### Western blotting

Western blot was conducted as previously described [31]. Briefly, the RIPA lysis buffer was used to harvest cells. 30 μg of proteins were loaded and separated by sodium dodecyl sulfate–polyacrylamide gel electrophoresis (SDS-PAGE). After the proteins were transferred to the PVDF membranes, primary and secondary antibodies were used to probe the target protein, which would then be visualized using enhanced chemiluminescence (Millipore, Bedford, MA). The primary antibodies included anti-β-actin (A5441; Sigma-Aldrich) and anti-IMMT (PA3-870, Thermo Fisher Scientific, Waltham, MA, USA). The signal of β-actin served as a loading control. The blotting signal intensity was quantified by GelQuant.NET v1.8.2 (Accessed from http://biochemlabsolutions.com/).

### Wound healing assay

25,000 cells were seeded on a 24-well plate 1 day prior to the siRNA treatment. 72 h after transfection, a wound was scratched in each well using a 10 μl pipette tip. Cell debris was washed twice with 1×PBS and then cultivated in a fresh medium. Wound closure was monitored at 0 and 22 h on a Lionheart FX microscope (Agilent Technologies, CA, USA). The quantification of the wound area was determined by ImageJ.

### Cell viability analysis

The colorimetry-based Cell Counting Kit-8 (CCK-8) (96,992; Sigma-Aldrich) was used to evaluate cell viability. 10,000 per 100 μL of MDA-MB-231 cells were seeded into a 96-well plate. After 18 h, the attached cells were then transfected with si-ctrl or si-IMMT for 72 h. The CCK-8 solution was then added to the medium for 1 h at 37 °C. The colorimetric signal was acquired at a wavelength of 450 nm on a microplate (Hidex Sense microplate reader, Turku, Finland). The reference wavelength was set at 630 nm.

### Flow cytometry

Cell cycle analysis was conducted as described in a previous report [31]. Briefly, ice-cold 95% ethanol was used to fix the cell. 10 μg/mL of propidium iodide was used to probe the DNA content. The signal representing the DNA content was then acquired by an LSR II flow cytometer (BD Biosciences, Franklin Lakes, NJ, USA). To detect mitochondrial membrane potential (MMP), cells were incubated with 100 nM TMRM (Tetramethylrhodamine Methyl Ester Perchlorate) (T668; Thermo Fisher Scientific). After PBS washing, the fluorescence signal was examined on an LSR II flow cytometer. To measure reactive oxygen species (ROS), MitoSOX™ Red (M36008; Thermo Fisher Scientific) and CM-H_2_DCFDA (C6827, Thermo Fisher Scientific) were employed to detect mitochondrial ROS and intracellular general ROS, respectively. 5 μM of MitoSOX™ Red and CM-H_2_DCFDA were respectively applied to cells, which were incubated for 30 min at 37 °C, after which fluorescence analysis was performed using an LSR II flow cytometer.

### Analysis of oxygen consumption rate (OCR)

Cellular OCR was evaluated using a Seahorse XF24 analyzer (Seahorse Bioscience, Billerica, MA) as previously described [32], adhering to the manufacturer’s instructions with minor modifications. Briefly, 50,000 cells were plated in a Seahorse Flux Analyzer plate. After 18 h, the plate was pre-heated at 37 °C for 1 h. We documented three measurements each of basal OCR, proton-leak OCR, and maximal OCR. The proton-leak OCR was assessed using 1 μM oligomycin. Maximal OCR was driven by treating the cells with 300 nM FCCP. Finally, non-mitochondrial respiration was obtained by injection of 1 μM rotenone.

### Ethics statement and specimen collection for immunohistochemistry

The clinical studies were performed in compliance with the approved guidelines of the Institutional Review Board (IRB) of Show Chwan Memorial Hospital (Approval Number: 1091208; approval date: December 8, 2020). The immunohistochemistry (IHC) assays and scoring methods were carried out based on previously established standard procedures [33].

### Investigation of potent drug candidates according to IMMT expression

The CRISPR-screen of the Genomics of Drug Sensitivity in Cancer (GDSC) data repository was used to analyze IMMT expression-based drug sensitivity [34]. Data regarding sensitivity to pyridostatin was retrieved from the CCLE [35].

### Statistical analyses

Statistical comparisons were conducted as previously described [36]. Briefly, unpaired t-test and one-way analysis of variance (ANOVA) were employed to analyze quantitative data of two-group and three-or-more-group comparisons, respectively. Pearson correlation coefficient was utilized to determine the relationship between two variables. Kaplan–Meier analysis and log‐rank test were carried out to compare the survival rate. Receiver operating characteristics (ROC) was used to evaluate the diagnostic efficiency of IMMT. The interconnectivity between the high expression of MICOS genes and the survival rate was illustrated using the chord diagram in the circlize package of RStudio Cloud (https://rstudio.cloud/content/yours?sort=name_asc).

## Results

### Clinicopathological screening of IMMT-centric MICOS components in BC

**Fig. 1:**
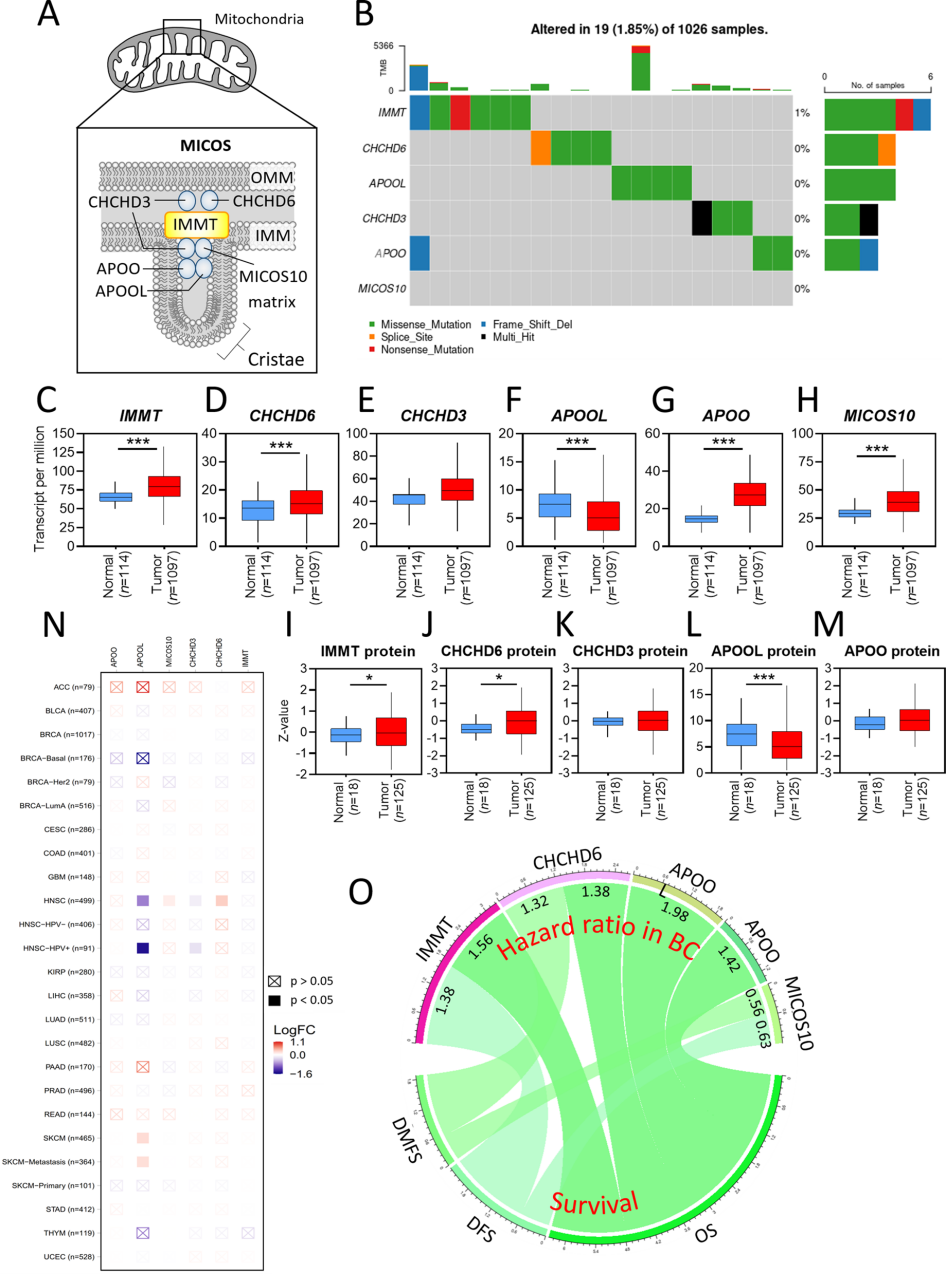
Clinicopathological Screening of IMMT-centric MICOS Components in BC. **A** Schematic diagram depicting the MICOS subunits in mitochondria. **B** Single nucleotide variant (SNV) oncoplot of BC samples. The number and percentage of distinct SNVs, including missense mutation (green), nonsense mutation (red), splice site (orange), frame shift/deletion (light blue) and presence of multiple types of mutation in the same gene (Multi Hit, black) on each gene in 1,026 BC samples are indicated. Level of tumor mutation burden (TMB) in each sample is documented on the top of the oncoplot. **C**–**H** the gene expression levels of IMMT (**C**), CHCHD6 (**D**), CHCHD3 (**E**), APOOL (**F**), APOO (**G**), and MICOS10 (**H**) in normal tissues and BC tumors. **I–M** the protein expression levels of IMMT (**I**), CHCHD6 (**D**), CHCHD3 (**E**), APOOL (**F**), APOO (**G**), and MICOS10 (**H**) in normal tissues and BC tumors accessed from the CPTAC repository. *p < 0.05, ***p < 0.001 between the two groups. **N** heatmap comparing the differential gene expressions of IMMT, CHCHD6, CHCHD3, MICOS10, APOOL, and APOO between different mutation statuses. LOG FC, fold change expressed as log logarithm to the base 10. **O** Chord diagram illustrating the relationship between the hazard ratio in BC patients harboring high gene expressions of IMMT, CHCHD6, APOOL, APOO, and MICOS10 and survival rate. *OS* overall survival; *DFS* disease-free survival, *DMFS* distant metastasis free survival

We first examined the genetic variant profiles of MICOS subunits in BC samples of the TCGA repository (Fig. 1A). 19 out of 1,026 BC samples (1.85%) showed an altered single nucleotide variant (SNV). IMMT showed the highest SNV counts (6 samples) compared to CHCHD6 (4 samples), CHCHD3 (3 samples), APOO (3 samples), APOOL (4 samples), and MICOS10 (0 samples) (Fig. 1B). The majority of the SNVs were missense mutations (Fig. 1B). In the TCGA datasets, BC tumor tissue showed elevated gene expression levels of IMMT, CHCHD6, CHCHD3, APOO, and MICOS10 as compared to normal tissue, while the APOOL level was decreased in tumor tissue as compared to normal tissue (Fig. 1C–H). We subsequently examined the protein level expression by accessing CPTAC datasets. The protein expressions of IMMT, CHCHD6, CHCHD3, and APOOL followed a similar trend to the gene expressions, except for APOO (Fig. 1I–M). The comparison of the different mutation status between the differential gene expressions revealed that the mutations of IMMT, CHCHD6, CHCHD3, APOO, and *MICOS10* demonstrated no significant associations with gene expressions (Fig. 1N). The log-rank test was used to analyze the association between gene expressions and survival rates. As shown in Fig. 1O (all *p* < 0.05), high *IMMT* expression was associated with an unfavorable overall survival (OS) and disease-free survival (DFS); high CHCHD6 expression was associated with an unfavorable OS and distant metastasis free survival (DMFS); high APOOL and APOO expressions were associated with an unfavorable OS; while high MICOS10 expression was associated with favorable DMFS and DFS.

### Differential expression and diagnostic value of IMMT in BC

**Fig. 2:**
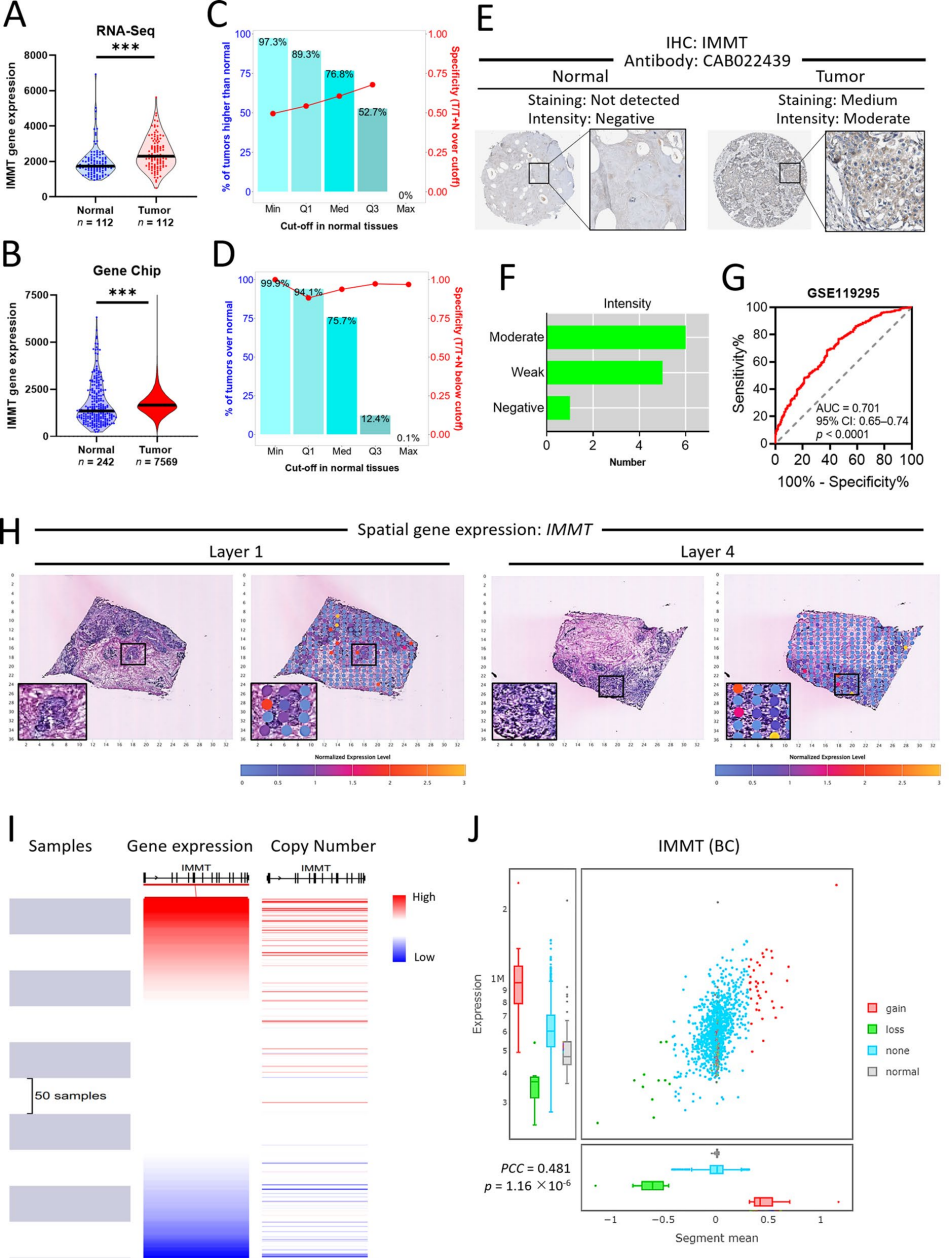
Differential expression and diagnostic value of IMMT. **A** and **B** violin plots showing the IMMT expression levels determined by RNA-seq (**A**) or gene chip (**B**) in normal and BC tumor tissues. ***p < 0.0001 between indicated groups. **C** and **D** the percent and specificity at various cut-off points in normal tissue from RNA-seq data (**C**) and gene chip data (**D**). **E** and **F** IHC staining for the detection of IMMT protein expression levels by antibody CAB022439 in normal and BC tissues (**E**). The number of BC patients (n = 11) determined with moderate, weak, or negative staining intensity. **G** ROC curves for normal and BC cohorts based on IMMT levels from the GSE119295 dataset. **H** Spatial IMMT expression in human BC tissue sections as determined by spatial transcriptomics. Black square enlarging the dark-staining tissue that indicates a tumor nest composed of hyperchromatic cancer cells. **I** and **J** the correlation of IMMT gene expression with its copy number. Heatmap analyzed o visualizing the expression-copy number relationship of GDC-TCGA Breast Cancer dataset (**I**). Pearson correlation coefficient (PCC) of tumors with IMMT copy number status of gain, loss, and none, and normal tissue based on TCGA breast cancer datasets

**Fig. 3:**
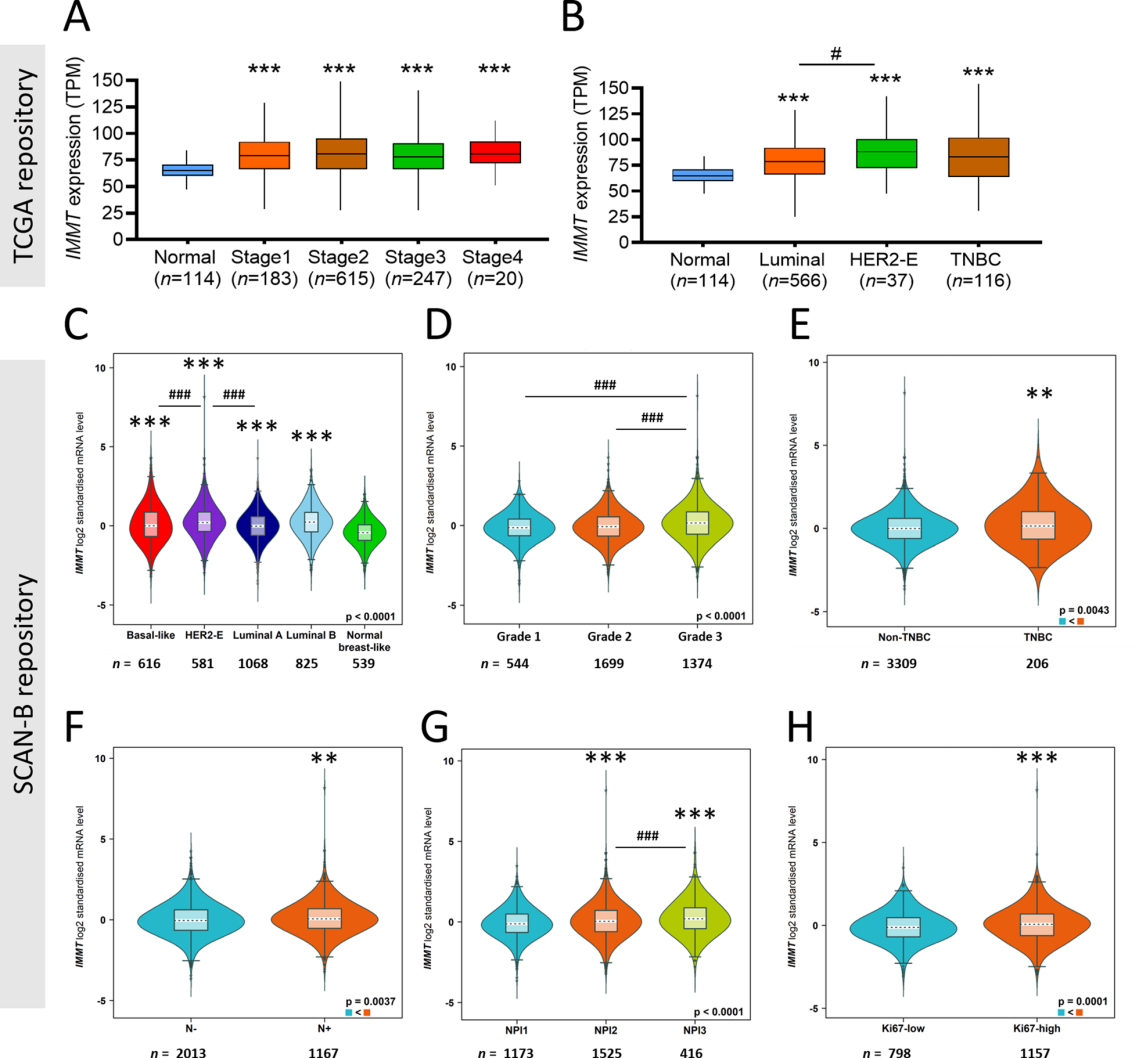
IMMT expression according to clinical factors and pathological categories from multiple databases. **A** and **B** The Cancer Genomic Atlas (TCGA) repository-based IMMT expression levels. Box plot of IMMT expression according to clinical stages (**A**) and major subtypes (**B**). TPM, Transcripts Per Million. **C**–**H** SCAN-B (The Sweden Cancerome Analysis Network-Breast) repository-based IMMT expression levels. Violin plot of IMMT expression according to PAM subtypes (**C**), SBR (Scarff Bloom and Richardson) grade (**D**), IHC status of TNBC (triple-negative breast cancer) (**E**), nodal status (**F**), NPI (Nottingham prognostic index) (**G**) and IHC status of Ki67 (H). **p < 0.01, ***p < 0.001 when compared with normal group. N^−^, NPI1, or Ki67 low groups. ^#^p < 0.05, ^###^p < 0.001 when comparing indicated groups

Both the RNA-seq data and chip array data revealed that *IMMT* expression levels were increased in BC tumor tissue as compared to normal tissue (Fig. 2A, B). The specificity of RNA-seq data increased with a higher cut-off value in normal tissue, while the chip array data sustained a high specificity regardless of the cut-off point (Fig. 2C, D). The immunohistochemistry staining data of the Human Protein Atlas confirmed an overexpressed IMMT protein in BC tumor tissue compared to normal tissue (Fig. 2E–F). We constructed an ROC curve to evaluate the diagnostic efficiency of IMMT for BC based on GSE11925 datasets. We noted that the IMMT expression levels demonstrated a high diagnostic accuracy (AUC: 0.701, 95% CI 0.65–0.74, *p* < 0.0001) (Fig. 2G). Spatial transcriptomic analysis of IMMT showed that a high level of IMMT was concurrently expressed with the cancerous section of BC tumor tissue (Fig. 2H). UCSC Xena analysis and TCGA-BRCA repository data demonstrated that the IMMT expression level closely correlated with the copy number (Fig. 2I, J**)**. We then investigated the IMMT expression based on various clinical categories using different databases, including TCGA and The Sweden Cancerome Analysis Network-Breast (SCAN-B) [37]. BC tumors of all clinical stages showed higher IMMT levels than normal tissue (Fig. 3A). The TCGA repository data indicated that all BC subtypes, including luminal, HER2-enriched (HER2-E), and triple-negative breast cancer (TNBC) showed increased IMMT levels, while HER2-E had higher IMMT levels than luminal (Fig. 3B). Similarly, the SCAN-B repository data indicated that all BC subtypes had higher IMMT than normal, while HER2-E had higher IMMT than basal-like and luminal A (Fig. 3C). Grade 3 tumors showed increased IMMT compared to those of grades 1 or 2 (Fig. 3D). A TNBC tumor had increased IMMT as compared to a non-TNBC tumor (Fig. 3E). Nodal status positive (N^+^) showed higher IMMT levels compared to nodal status negative (N^–^) (Fig. 3F). IMMT levels were increased with an elevated Nottingham prognostic index (NPI) score (Fig. 3G). Additionally, we examined the IMMT expression based on the Ki67 staining levels with IHC, which is an independent prognostic biomarker for BC [38]. As shown in Fig. 3H, the Ki67-high tumor had a higher IMMT level than the Ki67-low tumor. Collectively, these results demonstrate that IMMT expression may serve as a diagnostic marker and that IMMT is linked to an advanced disease status.

### Prognostic value of IMMT

**Fig. 4:**
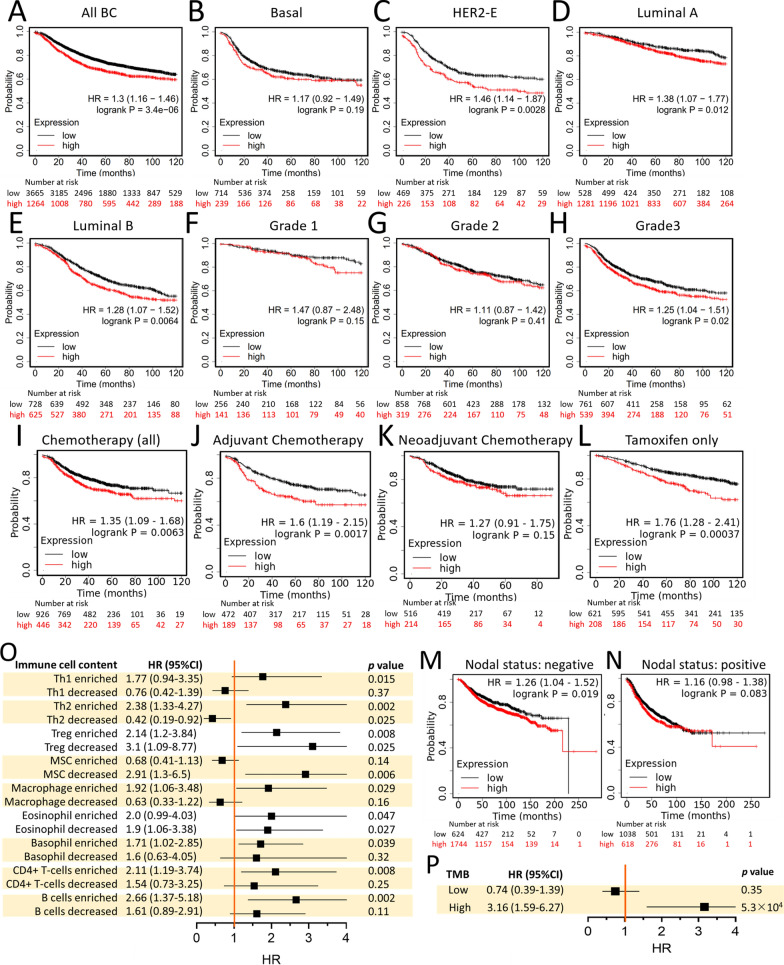
Prognostic value of IMMT in BC. **A**–**N** Kaplan–Meier survival analysis of relapse-free survival (RFS) based on IMMT expression. “Auto select best cut off” algorithm function was used to divide low/high groups in all BC patients (**A**), PAM50 subtype with basal (**B**), HER2-E (HER2-enriched) (**C**), luminal A (**D**) and luminal B (**E**), grade 1 (**F**), grade 2 (**G**), grade 3 (**H**), patients undergoing any chemotherapy (**I**), patients undergoing adjuvant chemotherapy (**J**), patients undergoing neoadjuvant chemotherapy (**K**), patients undergoing tamoxifen only (**L**), nodal status negative (**M**), and nodal status positive (**N**). **O** and **P** forest plot illustrating the HR of high IMMT, the 95% CI when harboring various immune cell contents (**O**) and TMB extent (**P**)

To gain more insight into prognosis, we examined the correlation between IMMT expression and survival rate. In this regard, recurrence-free survival (RFS) was considered as an appropriate indicator of the occurrence of metastasis. High IMMT expression in BC patients was found to be associated with decreased RFS time (Fig. 4A). More specifically, IMMT acted as a prognostic indicator in HER2-E, luminal A and luminal B, although not in basal type (Fig. 4B–E). A high IMMT expression indicated decreased RFS time in BC patients with grade 3, while no differences were noted in grades 1 and 2 (Fig. 4F–H). In terms of therapeutic scenarios, a high IMMT expression indicated decreased RFS time in BC patients undergoing all types of chemotherapy, adjuvant chemotherapy or tamoxifen, but not for neoadjuvant chemotherapy (Fig. 4I–L). In addition, IMMT can serve as an independent factor in N^–^, but not in N^+^ (Fig. 4M, N).

As the tumor-infiltrating immune cells (TIICs) are indispensable parts of the TIME and implicated in clinical outcome [39], we then looked into the prognostic value of IMMT in BC when considering the immune cell constituents. High IMMT did not statistically indicate decreased RFS time in BC tumors harboring decreased helper T cell type 1 (Th1), decreased Th2, enriched mesenchymal stem cell (MSC), decreased macrophage, decreased basophil, decreased CD4 + T cell, and decreased B cell (Fig. 4O). It is worth noting that high IMMT indicated decreased RFS time in BC tumors harboring a high tumor mutation burden (TMB), but not in those with a low TMB (Fig. 4P). Collectively, high IMMT present a prognostic prediction of an unfavorable outcome in BC patients, particularly in subtype HER-2E, luminal A and luminal B, tumor grade 3, treatment with adjuvant chemotherapy and tamoxifen, and in N^−^. However, the contents of Th1, Th2, MSC, macrophage, CD4 + T cell, B cell, and TMB can counteract the predictive efficacy.

### Single-cell level analysis

**Fig. 5:**
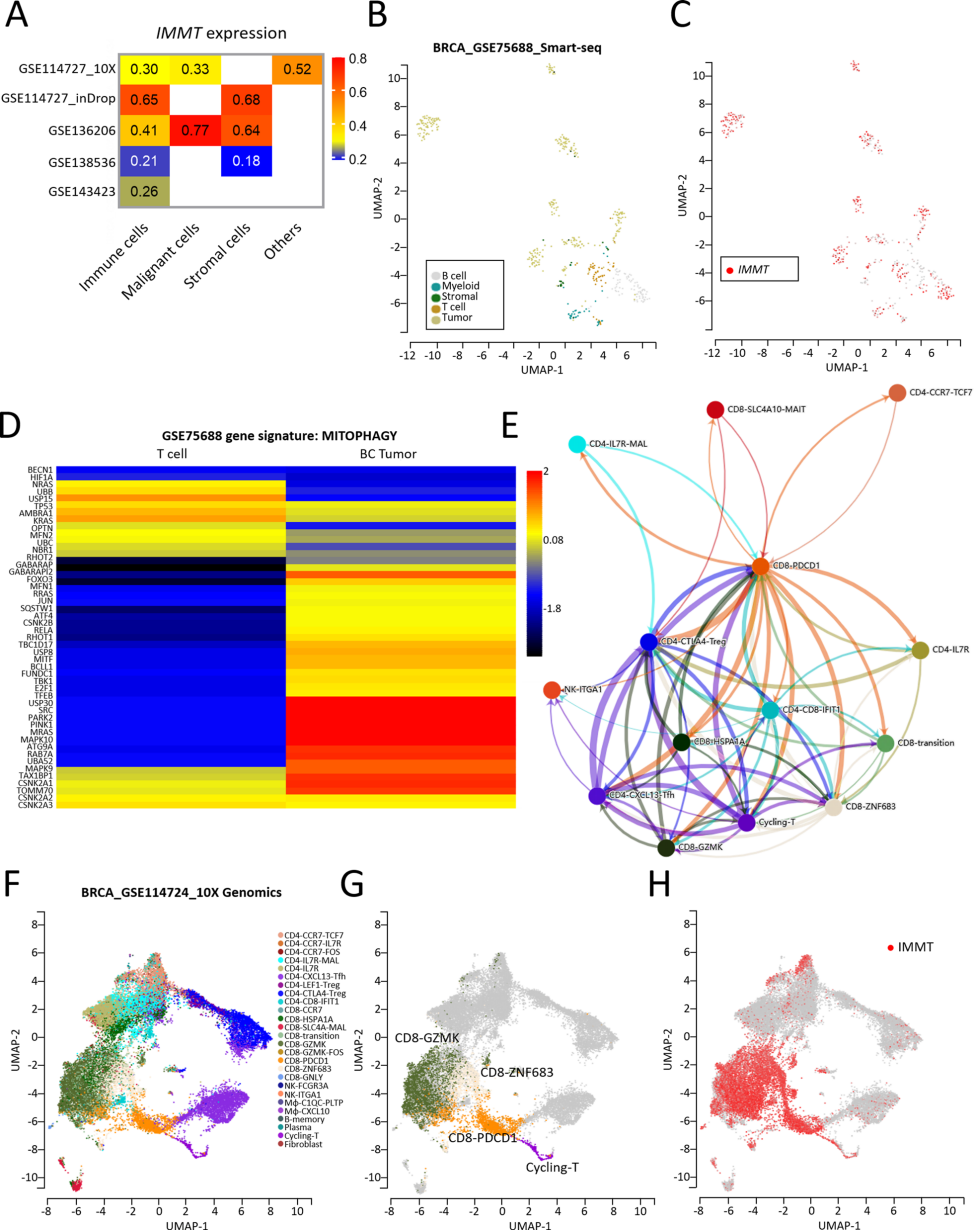
Analysis of IMMT at the single-cell level. **A**–**H** single-cell analysis. Heatmap showing the IMMT expression profile in immune cells, malignant cells, stromal cells and others of BC based on multiple GEO datasets (**A**). Uniform Manifold Approximation and Projection (UMAP) plots showing IMMT expression mapping on to different cell types in BC based on the GSE75688 dataset (**B** and **C**). Heatmap visualizing the gene signature MITOPHAGY of T cells and BC tumor (**D**). Communication network among immune cells (**E**). UMAP plots showing IMMT expression clusters (**F**–**H**)

We subsequently attempted to localize IMMT at the single-cell level. A TISCH-based single-cell analysis of BC based on multiple GEO datasets confirmed the expression of IMMT in immune cells, malignant cells, and stromal cells (Fig. 5A). The single-cell RNA-seq based on GSE75688 then showed that IMMT was predominantly expressed in tumor cells, although was relatively low in T cells (Fig. 5B, C**)**. Interestingly, the BC tumor was more abundant in gene signatures of mitophagy (Fig. 5D), in which IMMT acts as a key regulator [10]. Cellular communication retrieved from the scTIME Portal revealed complex interactions among various T cell subpopulations (Fig. 5E). The single-cell RNA-seq of GSE114724 revealed a positive correlation between a particular group of CD8 T cells (CD8-GZMK, ZNF683, PDCD1 and cycling-T) and IMMT expression (Fig. 5F–H).

### IMMT over-expression is associated with immunosuppressive TIME

**Fig. 6:**
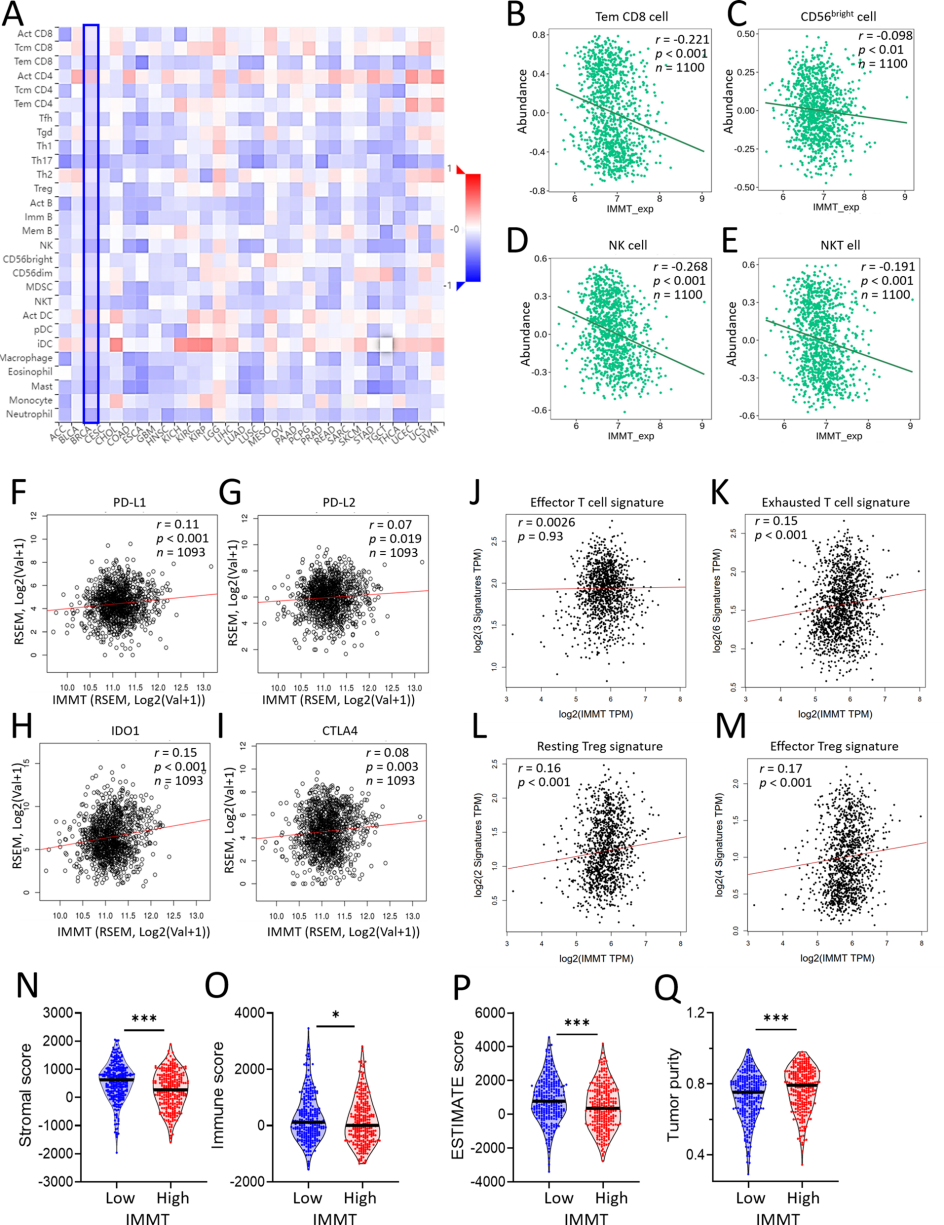
The role of IMMT in shaping TIME. **A**–**M** immunological analysis on immune infiltration and immunosuppressors. Heatmap showing the correlation between IMMT expression and lymphocytes infiltration across human cancers (**A**). Dot plots showing the correlation of IMMT with anti-tumor cell population such as effector memory CD8 T cell (Tem_CD8) (B), CD56^bright^ killer cells (**C**), NK cells (**D**), NKT cells (**E**), with immunosuppressive molecules such as PD-L1 (**F**), PD-L2 (**G**), IDO1 (**H**), and CTLA4 (**I**), and with effector T cell signature (CX3CR1, FGFBP2 and FCGR3A) (**J**), with exhausted T cell signature (HAVCR2, TIGIT, LAG3, PDCD1, CXCL13 and LAYN) (**K**), with resting Treg cell signature (FOXP3 and IL2RA) (**L**), and with effector Treg cell signature (FOXP3, CTLA4, CCR8 and TNFRSF9) (**M**). Violin plots showing the stromal score (**N**), immune score (**O**), EXTIMATE score (**P**) and tumor purity (**Q**). *p < 0.01, **p < 0.01, and ***p < 0.001 between the low and high IMMT groups

Considering the vital role of TIME in tumor growth, spread, and escape from immune-mediated destruction [40], we analyzed the associations between IMMT and tumor-immune interaction. A Spearman correlation analysis of TISIDB revealed that IMMT expression negatively correlated with most tumor-infiltrating lymphocytes in BC (Fig. 6A), such as effector memory CD8 T cells (Tem_CD8) (Fig. 6B), CD56^bright^ killer cells (Fig. 6C), natural killer (NK) cells (Fig. 6D), and natural killer T (NKT) cells (Fig. 6E). Moreover, we observed that IMMT has a positive association with T cell-suppressors, such as PD-L1 (Programmed Cell Death 1 Ligand (1) (Fig. 6F), PD-L2 (programmed cell death 1 ligand 2) (Fig. 6G), IDO1(indoleamine 2,3-dioxygenase (1) (Fig. 6H), and CTLA4 (cytotoxic t-lymphocyte associated protein (4) (Fig. 6I). A Pearson correlation analysis conducted using GEPIA2 demonstrated that IMMT had no significant correlation with the effector T cell signature (Fig. 6J), while showing a significant positive correlation with the exhausted T cell signature (Fig. 6K), resting Treg cell signature (Fig. 6L) and effector Treg cell signature (Fig. 6M). Furthermore, the ESTIMATE analysis [23] demonstrated that the cohort with high IMMT exhibited a reduced stromal score (Fig. 6N), immune score (Fig. 6O) and ESTIMATE score (Fig. 6P), while showing enhanced tumor purity (Fig. 6Q). Collectively, these results indicate that IMMT over-expression is associated with an immunosuppressive TIME.

### Involvement of IMMT in cell cycle progression and mitochondrial antioxidant defenses

**Fig. 7:**
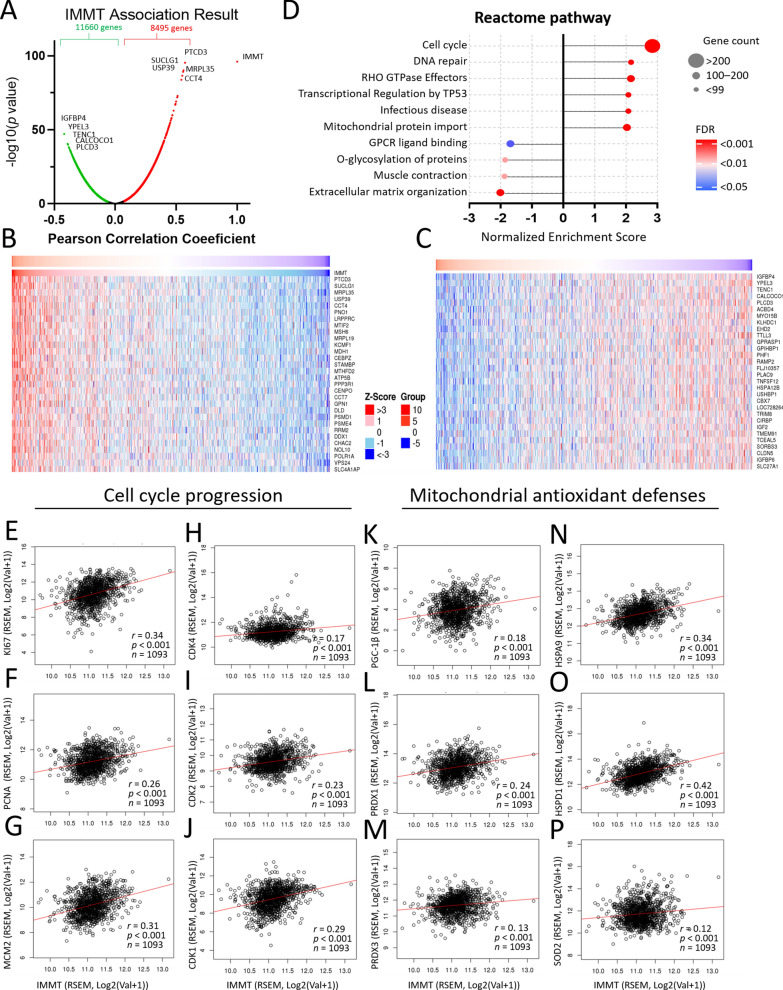
Involvement of IMMT in cell cycle progression and mitochondrial antioxidant defenses. Genetic and enrichment analyses of TCGA samples were conducted using the LinkedOmics functional modules. Volcano plot showing the PCC and p value of the IMMT-coexpressed genes (**A**). Heatmap showing top 30 positively (**B**) and negatively (**C**) correlated genes with IMMT. Bubble plot showing the normalized enrichment score, involved gene count and false discovery rate (FDR) of the Reactome pathway terms (**D**). Dot plots showing the correlation between IMMT and Ki67 (**E**), PCNA (**F**), and MCM2 (**G**), CDK4 (**H**), CDK2 (**I**) and CDK1 (**J**), PGC-1β (**K**), PRDX1(**L**), PRDX3(**M**), HSPA9(**N**), HSPD1(**O**), and SOD2(**P**)

We further utilized Linkedomics [30] to analyze the functional enrichment of IMMT. A total of 8,495 genes had significant positive associations with IMMT, while 11,660 genes had significant negative associations (Fig. 7A). A heatmap visualized the top 30 individual genes (Fig. 7B, C). The gene set enrichment analysis (GSEA) revealed that genes co-expressed with IMMT were involved in the activation of the following Reactome pathway terms: cell cycle, DNA repair, RHO GTPase effectors, transcriptional regulation by TP53 infectious disease, and mitochondrial protein import (Fig. 7D). Meanwhile, the inhibited terms included: GPCR ligand binding, O-glycosylation of proteins, muscle contraction, and extracellular matrix organization (Fig. 7D). Notably, the enriched Reactome pathway terms showed that cell cycle has the highest Normalized Enriched Score (NES), the most gene counts and the lowest false discovery rate (FDR) (Fig. 7D). Considering the predominant impact of IMMT on cell cycle activity, we examined its association with critical biomarkers of cancer progression. As shown in Fig. 7E–J, IMMT levels positively correlated with biomarkers linked to cancer proliferation (KI67, PCNA, and MCM2) and with key regulators of the cell cycle (CDK4, CDK2 and CDK1). Due to the pivotal role of IMMT in regulating the redox status [41], we investigated its association with mitochondrial antioxidant defenses in BC tumor samples. IMMT had a positive association with PGC-1β (proliferator-activated receptor-γ coactivator 1 beta) (Fig. 7K), PRDX1 (peroxiredoxin 1) (Fig. 7L), PRDX3 (peroxiredoxin 3) (Fig. 7M), HSPA9 (heat shock protein family A member 9) (Fig. 7N), HSPD1 (heat shock protein family D member 1) (Fig. 7O), and SOD2 (superoxide dismutase 2) (Fig. 7P). These results suggest that IMMT may play a role in BC cell cycle progression by regulating mitochondrial redox status.

**Fig. 8:**
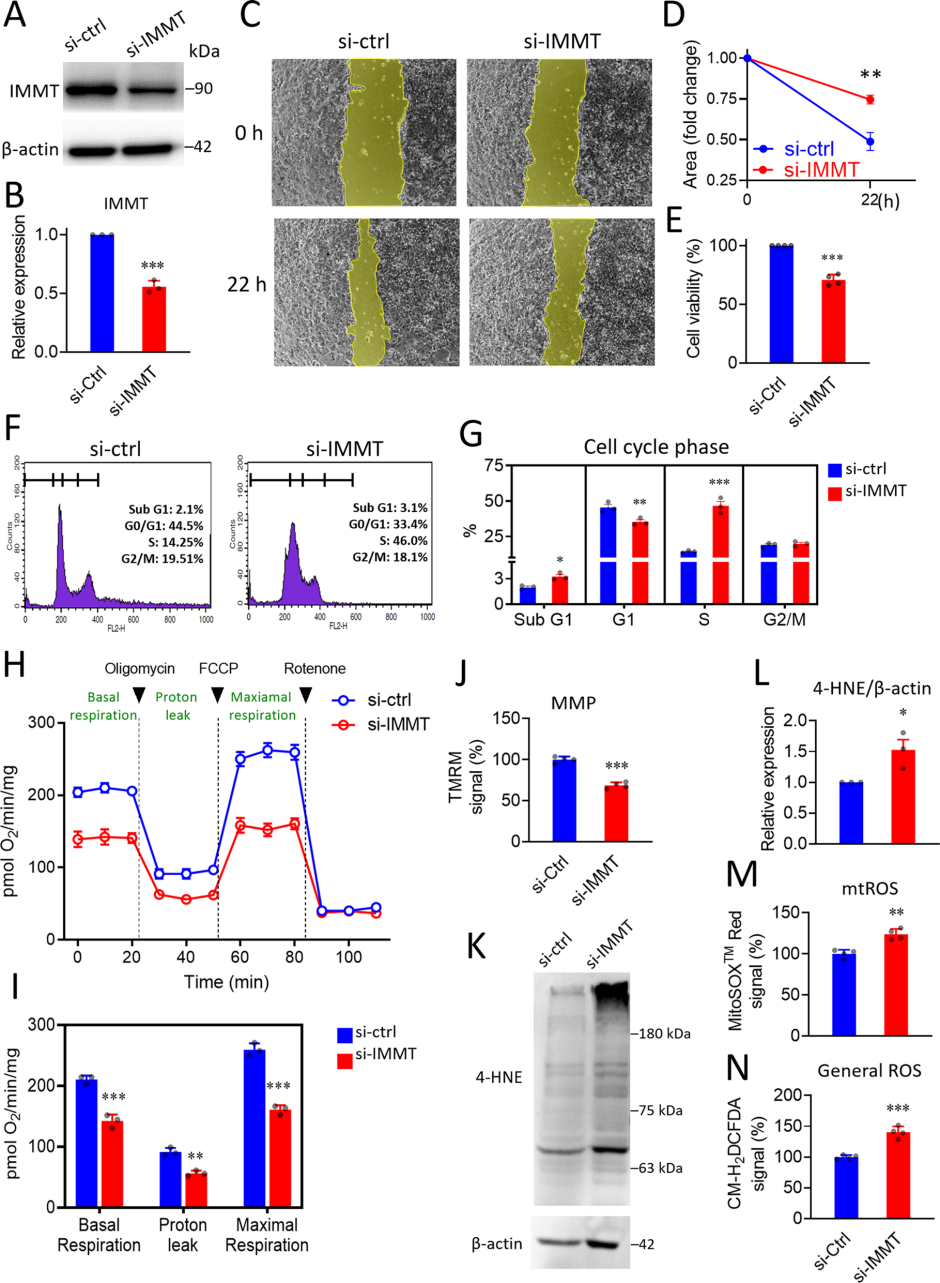
Knockdown of IMMT arrests the cell cycle and induces oxidative stress in BC cells. **A**–**G** Experimental verification of the biological role of *IMMT* on cancer cell biology. Representative western blot of IMMT in MDA-MB-231 cells treated with 20 nM negative control siRNA (si-ctrl) and IMMT siRNA (si-IMMT) for 72 h. β-actin serving as loading control (**A**). Bar chart showing the quantitative result of western blot (**B**). Representative images of wound healing assay at 0 h and 22 h (**C**). Quantification results of the wound area determined by the migrated cells (**D**). Cell viability evaluated by CCK-8 assay (**E**). Representative histograms of each cell cycle phase (**F**). Bar chart representing percentages of cell cycle phases (**G**). **H**–**N** Assessments of mitochondrial function and oxidative stress. Basal OCR (oxygen consumption rate), proton leak-OCR, and maximal OCR were measured in basal assay medium, 1 μM oligomycin, and 300 nM FCCP, respectively (**H**). Quantitative results of OCR (**I**). Mitochondrial membrane potential (MMP) determined by TMRM staining on a flow cytometer (**J**). Representative western blot of lipid peroxidation assessment by probing 4-HNE abundance. β-actin as loading control (**K**). Quantitative bar chart of 4-HNE abundance (**L**). The percentage of mitochondrial ROS (mtROS) and intracellular general ROS detected by MitoSOX^TM^Red (**M**), and CM-H_2_DCFDA (**N**), respectively. ***p < 0.001. **p < 0.01 and **p < 0.05

To verify the biological role of IMMT in BC, we conducted an siRNA-based knockdown in MDA-MB-231 cells (Fig. 8A–B). The wound healing assay revealed that cells undergoing IMMT-knockdown had reduced cell migration abilities and viability compared to cells subjected to negative control siRNA (Fig. 8C–E). In addition, knockdown of IMMT led to an increased proportion of sub-G1 phase, a decreased G1 phase, and an increased S phase (Fig. 8F, G). The respiration activity and mitochondrial membrane potential (MMP) were reduced by the knockdown of IMMT (Fig. 8H–J). Notably, the indicator of lipid peroxidation 4-Hydroxynonenal (4-HNE) was increased (Fig. 8K, L). Enhancements in mitochondrial ROS and general ROS were noted (Fig. 8M, N). Similarly, IMMT knockdown suppressed cell migration ability (Additional file 1: Fig. S1A, B) and induced lipid peroxidation (Additional file 1: Fig. S1C, D) in MCF-7 cells. Collectively, these results indicate that IMMT exerts positive roles in the regulation of mitochondrial fitness and intracellular oxidative stress, which may account for the motility and proliferative capacity of BC cells.

### Validation of the clinicopathological significance of IMMT in ethnic Chinese patients and from a pan-cancer perspective

**Fig. 9:**
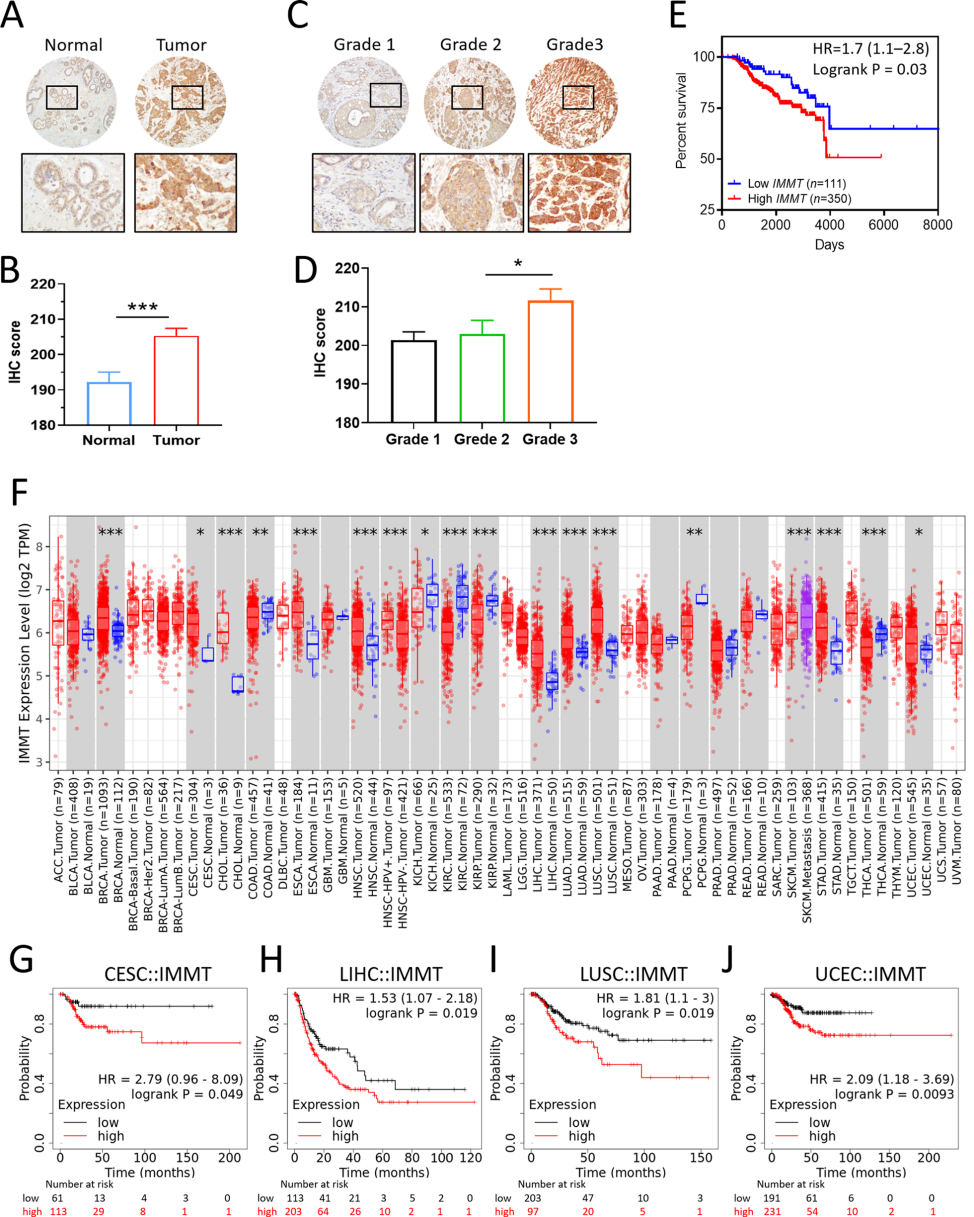
Validation of the clinical significance of IMMT in ethnic Chinese BC specimens and from a bioinformatic pan-cancer perspective. **A**–**E** High IMMT expression as a pathological biomarker and a prognostic factor for poor outcome in ethnic Chinese patients with BC. Representative image and quantitative bar charts of tumor-adjacent normal tissue vs. tumor (**A** and **B**) and grade 1 vs. grade 2 vs. grade 3 tissues (**C** and **D**). Note that the non-neoplastic mammary gland duct cells and malignant mammary gland duct cells show mild and strong cytoplasmic expression of IMMT, respectively (100x) (**A**). The grade 1, grade 2, and grade 3 invasive ductal carcinoma cells show mild, moderate, and strong cytoplasmic expressions of IMMT, respectively (**C**). Kaplan–Meier curve of the overall survival (cutoff value high/low: 67%/33%) (**E**). **F**–**J** Generalization value of IMMT across cancers. ***p < 0.0001, **p < 0.01, *p < 0.05 between tumor and normal (**F**). Kaplan–Meier curve representing the association between IMMT and RFS in cervical squamous cell carcinoma (CESC) (**G**), liver hepatocellular carcinoma (LIHC) (**H**), lung squamous cell carcinoma (LUSC) (**I**), and uterine corpus endometrial carcinoma (UCEC) (J)

To verify the clinical value of IMMT, we collected tumor tissues and the clinical documents of BC patients (*n* = 461) from our hospital. IHC staining showed that the tumor tissues expressed higher IMMT levels than the tumor-adjacent normal tissue (Fig. 9A, B), and that grade 3 tumor tissue expressed higher IMMT levels than grade 2 (Fig. 9C, D). The Kaplan–Meier analysis revealed that patients with a high IMMT IHC score had decreased OS time (Fig. 9E). Similar to the observed bioinformatics results, grade 3 patients with high IMMT had a shorter overall survival time compared to low IMMT, even if there is no statistical significance (Additional file 1: Fig. S2). In this regard, our clinical findings correspond with the multi-omics data.

To clarify whether IMMT has a generalization value, we examined the expression profile across cancers and their corresponding normal tissues. We noted that multiple cancer types showed similar expression patterns to BC, including cervical squamous cell carcinoma (CESC), cholangiocarcinoma (CHOL), esophageal carcinoma (ESCA), head-neck squamous cell carcinoma (HNSC), liver hepatocellular carcinoma (LIHC), lung squamous cell carcinoma (LUSC), stomach adenocarcinoma (STAD), and uterine corpus endometrial carcinoma (UCEC) (Fig. 9F). High IMMT had significant associations with shorter RFS time in CESC (Fig. 9G), LIHC (Fig. 9H), LUSC (Fig. 9I), and UCEC (Fig. 9J).

### Pharmacogenetic analysis identified pyridostatin as a potent drug for high IMMT expression

**Fig. 10:**
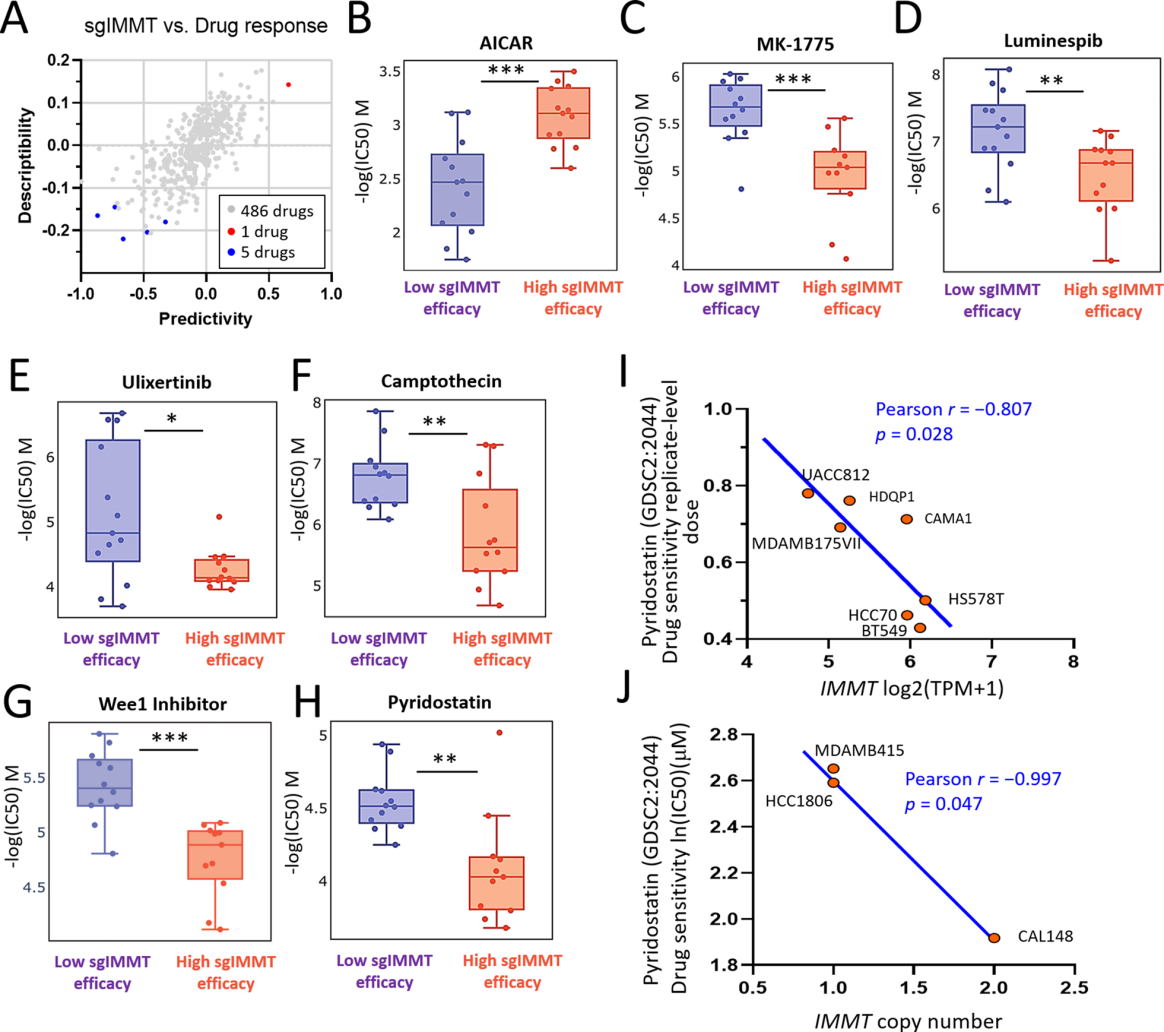
Pharmacogenetic analysis identified pyridostatin as a potent drug for high IMMT expression. **A**–**J** Identification of pyridostatin as a potential drug candidate. Scatter plot showing scores of predictivity and descriptivity for drugs acting on BC cells with various single guide IMMT (sgIMMT) efficacy. Dots in red and blue represent hits with a predictivity p-value of < 0.05 and a descriptivity p-value of 0.05 (**A**). Boxplots showing –log (IC50) M of AICAR (5-Aminoimidazole-4-carboxamide ribonucleotide) (**B**), MK-1775(**C**), luminespib (**D**), ulixertinib (**E**), camptothecin (**F**), wee1 inhibitor (**G**), and pyridostatin (**H**). *p < 0.05, **p < 0.01, ***p < 0.001 between indicated groups. Dot plots showing the correlation of pyridostatin sensitivity with IMMT expression (**I**) and with IMMT copy number (**J**)

To explore potentially effective pharmaceutical agents targeting BC, we conducted cross-association analyses between drug response and IMMT knockdown using single-guide RNA (sgRNA)-mediated CRSPR in BC cells. Among the 486 screened drugs, 6 drugs were identified to exert altered potency (Fig. 10A). BC cell lines with high sgIMMT efficiency exhibited increased sensitivity to AICAR (Fig. 10B). Meanwhile, BC cell lines with low sgIMMT efficiency exhibited increased sensitivity to MK-1775 (Fig. 10C), luminespib (Fig. 10D), ulixertinib (Fig. 10E), camptothecin (Fig. 10F), wee1 inhibitors (Fig. 10G), and pyridostatin (Fig. 10H). Moreover, CCLE datasets confirmed the relationship between pyridostatin and IMMT expression. The pyridostatin sensitivity correlated negatively with IMMT expression levels (Fig. 10I) and IMMT copy numbers (Fig. 10J). The analyses revealed that pyridostatin exerts a potentiation effect when cells exhibit a high expression of IMMT, indicating that it may be a viable candidate for the development of precision medicine.

## Discussion

**Fig. 11:**
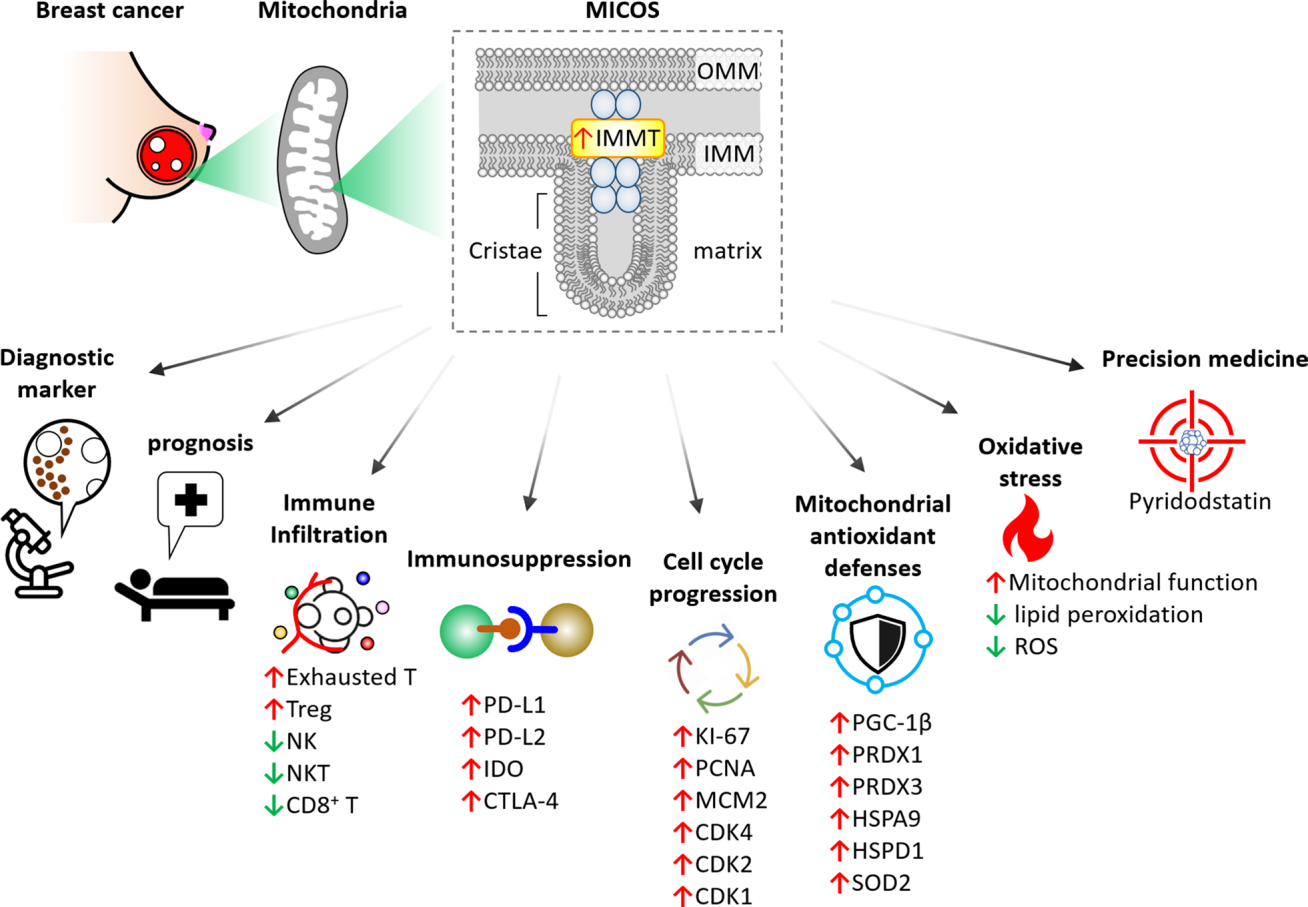
Schematic model delineates IMMT’s relevance in diagnosis, prognosis, immuno-oncology, cell cycle progression, mitochondrial antioxidant defenses, oxidative stress, and potential precision treatments for BC

The physiological function of IMMT in regulating mitochondrial dynamics and preserving mitochondrial structural integrity is an emerging focus of research; meanwhile, its clinicopathological value, association with TIME, and therapeutic implications in patients with BC have yet to be clarified. In this study, we combined multi-omics analysis, clinical validation and cellular experiments to reveal the novel role of IMMT. BC with high IMMT expression serves as an independent diagnostic biomarker, correlates with advanced clinical status, and predicts poor outcome. In addition, we reveal here that the contents of Th1, Th2, MSC, macrophages, basophil, CD4 + T cell and B cell, and TMB levels can counteract the prognostic significance. Our analyses at the single-cell level and of whole tissue samples demonstrate that elevated IMMT is associated with an immunosuppressive TIME. The potential mechanism of IMMT overexpression underlying BC progression may lie in the co-expressed genes implicated in cell cycle progression and mitochondrial antioxidant defenses. Genetic manipulation confirmed that IMMT plays a positive role in mitochondrial function and oxidative stress. Moreover, we demonstrate that the clinicopathological values of IMMT are amenable to ethnic Chinese BC patients and can be generalized to other cancer types, including CESC, LIHC, LUSC, and UCEC. Of particular note, we here identify pyridostatin as an effective drug candidate when BC cells are harboring elevated IMMT expression, thereby offering a promising therapeutic candidate for precision medicine. A schematic model of IMMT’s relevance in the clinical setting, immuno-oncology, redox biology, and precision medicine is illustrated in Fig. 11.

Limited information regarding the clinicopathological significance of IMMT in breast cancer is currently available. Although, Suárez-Arroyo et al. have reported an upregulated IMMT protein level in BC cells compared to normal mammary epithelial cells [42]. Other cancer types, such as those in the liver, prostate, colon, and pancreas have also been reported to express altered IMMT levels [43]. Meanwhile, Sotgia et al. reported that high IMMT was associated with an unfavorable outcome in gastric cancer [44]. In this study, IMMT over-expression was noted in BC tissue, and correlated with a shorter RFS in the patient group. High IMMT is associated with several statuses indicating a poor prognosis, including advanced grade, TNBC subtype, nodal status positive, high NPI score, and high KI-67 level.

As disorganization of the mitochondrial structure may result in activation of the immune response via leaked mitochondrial genome [45, 46], IMMT may play a role in the immune response. Indeed, Ghosh et al. have demonstrated that IMMT knockdown in cancer cells causes a catastrophic collapse of mitochondrial integrity and the activation of mitochondrion-directed innate immunity [47]. The bioinformatic investigation in our study demonstrates that IMMT upregulation is associated with immunosuppressed TIME in BC. Nevertheless, it must be noted that a major caveat of the present study lies in the lack of experimental verification of the causal relationship between IMMT and immune signaling activation in BC. Further investigation is thus required to further elucidate the molecular activities involved.

IMMT has been reported as a requirement for tumor cell proliferation, including for osteosarcoma cells, prostate cancer cells, and BC cells [47]. IMMT-knockout cells exhibit dysregulated mitochondrial functions, leading to an arrested cell cycle, reduced cell proliferation, suppressed tumor growth, and increased apoptosis. Similarly, our study shows that the upregulation of IMMT is implicated in activating the cell cycle pathway and is positively associated with key biomarkers relevant to cell proliferation. We also found that IMMT-targeting siRNA hindered cell proliferation and migration.

Pyridostatin has been reported to exert an anti-tumor effect through its G-quadruplexes binding activity, and has been shown to exert a highly specific activity against BRCA1/2-deficient tumors [48]. Furthermore, pyridostatin activates the cytoplasmic STING signaling pathway in cancer cells. Our investigation indicates that pyridostatin could be a promising therapeutic drug candidate for BC harboring high IMMT expression levels. As such, pyridostatin is a suitable candidate for further therapeutic development in combination with genetic testing and immunotherapy.

In conclusion, our study demonstrates the novel diagnostic and prognostic significance of IMMT in BC and reveals its role in TIME and the cell cycle. Moreover, the identification of pyridostatin, based on IMMT expression, offers promise for the development of a precision medicine strategy.

## Supplementary Information

**Additional file 1: Figure S1.** IMMT knockdown inhibits migration and induces lipid peroxidation in MCF-7 cells. Representative images of wound healing assay at 0 h and 22 h (**A**). Quantification line plot of the wound area determined by the migrated cells (**B**). *p < 0.05. Representative western blot of lipid peroxidation assessment by probing 4-HNE abundance. β-actin as loading control (**C**). Quantitative bar chart of 4-HNE abundance (**D**). **Figure S2.** Kaplan-Meier analysis of overall survival probability based on low/high IHC score of IMMT in BC patients with grade 3.

## Data Availability

The dataset supporting the conclusions of this article is included within the article.
