# Supplementary material for: Multi-omics and experimental analysis unveil theragnostic value and immunological roles of inner membrane mitochondrial protein (IMMT) in breast cancer
Source: J Transl Med. 2023 Mar 10;21:189. doi: 10.1186/s12967-023-04035-4 (PMC9999521; doi:10.1186/s12967-023-04035-4)
Supplement: Supplementary file 1 — Additional file 1: Figure S1. IMMT knockdown inhibits migration and induces lipid peroxidation in MCF-7 cells. Representative images of wound healing assay at 0 h and 22 h (A). Quantification line plot of the wound area determined by the migrated cells (B). *p < 0.05. Representative western blot of lipid peroxidation assessment by probing 4-HNE abundance. β-actin as loading control (C). Quantitative bar chart of 4-HNE abundance (D). Figure S2. Kaplan-Meier analysis of overall survival probability based on low/high IHC score of IMMT in BC patients with grade 3. [file 12967_2023_4035_MOESM1_ESM.docx]

**Multi-omics and Experimental Analysis Unveil Theragnostic Value and Immunological Roles of Inner Membrane Mitochondrial Protein (IMMT) in Breast Cancer**

Hung-Yu Lin, Hsing-Ju Wu, and Pei-Yi Chu


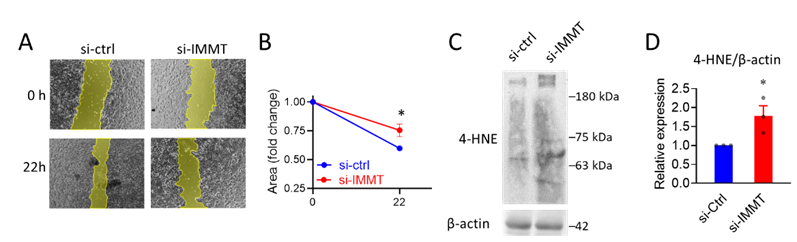


**Figure S1.** IMMT knockdown inhibits migration and induces lipid peroxidation in MCF-7 cells. Representative images of wound healing assay at 0 h and 22 h (A). Quantification line plot of the wound area determined by the migrated cells (B). *p < 0.05. Representative western blot of lipid peroxidation assessment by probing 4-HNE abundance. β-actin as loading control (C). Quantitative bar chart of 4-HNE abundance (D).


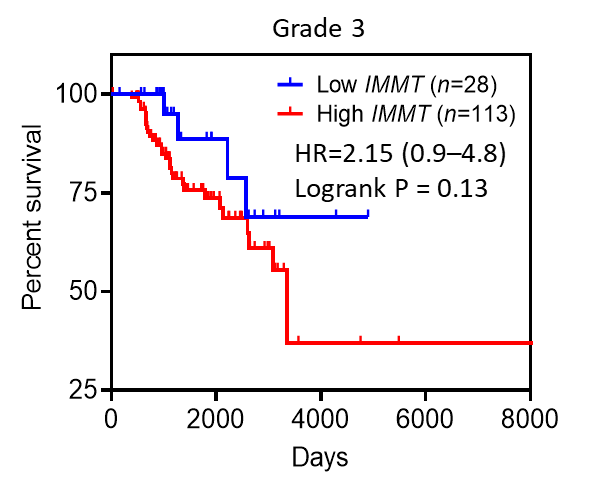


**Figure S2.** Kaplan-Meier analysis of overall survival probability based on low/high IHC score of IMMT in BC patients with grade 3.
